# Screening of Medicinal Herbs Identifies *Cimicifuga foetida* and Its Bioactive Component Caffeic Acid as SARS-CoV-2 Entry Inhibitors

**DOI:** 10.3390/v17081086

**Published:** 2025-08-05

**Authors:** Ching-Hsuan Liu, Yu-Ting Kuo, Chien-Ju Lin, Feng-Lin Yen, Shu-Jing Wu, Liang-Tzung Lin

**Affiliations:** 1 Department of Microbiology and Immunology, School of Medicine, College of Medicine, Taipei Medical University, Taipei 110, Taiwan; 2 Department of Diagnostic Imaging, Chi Mei Medical Center, Tainan 710, Taiwan; 3 Institute of Precision Medicine, College of Medicine, National Sun Yat-sen University, Kaohsiung 804, Taiwan; 4 Department of Radiology, School and College of Medicine, Kaohsiung Medical University, Kaohsiung 807, Taiwan; 5 School of Pharmacy, College of Pharmacy, Kaohsiung Medical University, Kaohsiung 807, Taiwan; 6 Department of Fragrance and Cosmetic Science, College of Pharmacy, Kaohsiung Medical University, Kaohsiung 807, Taiwan; 7 Drug Development and Value Creation Research Center, Kaohsiung Medical University, Kaohsiung 807, Taiwan; 8 Department of Medical Research, Kaohsiung Medical University Hospital, Kaohsiung 807, Taiwan; 9 Department of Nutritional Health, Chia-Nan University of Pharmacy and Science, Tainan 717, Taiwan; 10 Graduate Institute of Medical Sciences, College of Medicine, Taipei Medical University, Taipei 110, Taiwan

**Keywords:** SARS-CoV-2, medicinal herbs, herbal formulas, entry inhibitor, pseudoparticle, antiviral

## Abstract

The emergence of SARS-CoV-2 variants highlights the urgent need for novel therapeutic strategies, particularly entry inhibitors that could efficiently prevent viral infection. Medicinal herbs and herbal combination formulas have long been recognized for their effects in treating infectious diseases and their antiviral properties, thus providing abundant resources for the discovery of antiviral candidates. While many candidates have been suggested to have antiviral activity against SARS-CoV-2 infection, few have been validated for their mechanisms, including possible effects on viral entry. This study aimed to identify SARS-CoV-2 entry inhibitors from medicinal herbs and herbal formulas that are known for heat-clearing and detoxifying properties and/or antiviral activities. A SARS-CoV-2 pseudoparticle (SARS-CoV-2pp) system was used to assess mechanism-specific entry inhibition. Our results showed that the methanol extract of *Anemarrhena asphodeloides* rhizome, as well as the water extracts of *Cimicifuga foetida* rhizome, Xiao Chai Hu Tang (XCHT), and Sheng Ma Ge Gen Tang (SMGGT), have substantial inhibitory effects on the entry of SARS-CoV-2pps into host cells. Given the observation that *Cimicifuga foetida* exhibited the most potent inhibition and is a constituent of SMGGT, we further investigated the major compounds of the herb and identified caffeic acid as a bioactive component for blocking SARS-CoV-2pp entry. Entry inhibition of *Cimicifuga foetida* and caffeic acid was validated on both wild-type and the currently dominant JN.1 strain SARS-CoV-2pp systems. Moreover, caffeic acid was able to both inactivate the pseudoparticles and prevent their entry into pretreated host cells. The results support the traditional use of these herbal medicines and underscore their potential as valuable resources for identifying active compounds and developing therapeutic entry inhibitors for the management of COVID-19.

## 1. Introduction

Since its emergence in late 2019, SARS-CoV-2 has posed a significant global health threat, with over 777 million confirmed cases and over 7 million deaths reported as of December 2024 [1]. Although widespread vaccination has substantially mitigated disease severity and reduced COVID-19-related hospitalizations and mortality [2], the rapid evolution of SARS-CoV-2 has led to the emergence of multiple variants with increased transmissibility and immune evasion [3]. Studies have shown that vaccine effectiveness against infections and hospitalizations has declined over time, particularly in response to the Omicron variant [4,5]. As of June 2024, JN.1-lineage viruses have become dominant in circulation [6], and thus the U.S. FDA has recommended a monovalent JN.1-lineage-based vaccine formula for 2025–2026 [7]. As the virus continues to spread, there is an urgent need for ongoing vigilance in both preventive efforts and the development of effective therapeutic strategies to mitigate its impact.

Due to the limited availability of effective antivirals, COVID-19 management primarily focuses on symptomatic relief and supportive care. While agents like remdesivir, Paxlovid, and molnupiravir have received approval or emergency use authorization (EUA) [8], they have notable limitations: remdesivir requires intravenous administration, Paxlovid poses drug–drug interactions, and molnupiravir shows lower efficacy [9]. These challenges underscore the need for novel antiviral therapies with broad-spectrum activity, fewer drug interactions, and improved accessibility.

SARS-CoV-2 infection begins with viral attachment to host-cells’ surface receptors and co-receptors, including angiotensin-converting enzyme 2 (ACE2) [10], neuropilin-1 (NRP1) [11], CD147 [12], and HSPA5 (also known as GRP78) [13], mediated by the viral spike (S) glycoprotein. Viral entry occurs through either direct membrane fusion or endocytosis. Membrane fusion is facilitated by host serine protease TMPRSS2, which cleaves the spike protein at the S2′ site, exposing the fusion peptide and enabling viral–host membrane fusion [10,14], whereas endocytosis-mediated entry involves clathrin-dependent internalization, followed by endosomal acidification and spike cleavage by cathepsin L, leading to membrane fusion within the endosome [15,16]. Following entry, the viral genome is released into the cytoplasm and translated into polyproteins (pp1a and pp1ab), which are subsequently cleaved by viral proteases (3CL^pro^ and PL^pro^) into structural and non-structural proteins [17]. Given the critical role of viral entry in SARS-CoV-2 infection, developing entry inhibitors is a promising therapeutic strategy.

Natural products and herbal medicines have received considerable attention during the COVID-19 pandemic. Numerous candidates have been proposed for their antiviral and anti-inflammatory activities, and while some have shown promising effects [18,19,20,21], not all have undergone rigorous validation. Heat-clearing and exterior-releasing medicinal herbs, such as *Houttuynia Herba*, *Scutellaria Radix*, *Isatis Radix*, *Mori Folium*, and *Bupleurum Radix*, are widely used in traditional Chinese medicine and Japanese Kampo medicine for the treatment of COVID-19. These medicinal herbs could therefore provide a rich resource for the identification of antiviral entry inhibitors.

This study aims to evaluate the efficacy of medicinal herbal candidates in preventing SARS-CoV-2 entry and to identify candidate compounds within herbal matrices that may serve as the basis for future development of antiviral agents. We screened three panels of herbal extracts and herbal combination formulas for their ability to inhibit SARS-CoV-2 entry using pseudotyped viral particles bearing the SARS-CoV-2 spike protein. The active compound in the most effective herbal extract, *Cimicifuga foetida* water extract, was also identified and validated on both WT and JN.1 pseudoparticles for its antiviral effect and potential mechanisms.

## 2. Materials and Methods

### 2.1. Cell Culture

Human hepatoma Huh-7 cells were maintained in Dulbecco’s Modified Eagle’s Medium (DMEM; ThermoFisher Scientific, Waltham, MA, USA) supplemented with 10% fetal bovine serum (FBS; ThermoFisher Scientific), 1% gentamicin (ThermoFisher Scientific), and 1% amphotericin B (Sigma-Aldrich, Saint Louis, MO, USA). The 293FT cells were cultured in the same medium, supplemented with 0.5 mg/mL G418 sulfate (InvivoGen, San Diego, CA, USA) for selection. Cells were incubated at 37 °C in a 5% CO_2_ incubator. For all infection experiments, cells were cultured in DMEM containing 2% FBS with antibiotics as described above.

### 2.2. SARS-CoV-2 Pseudoparticle (SARS-CoV-2pp) Production

SARS-CoV-2 pseudoparticles (SARS-CoV-2pps) were produced as previously described [22]. The 293FT cells were seeded in 10 cm dishes and co-transfected with lentiviral vector and SARS-CoV-2 spike protein plasmids using the OMNIfect transfection reagent (Transomic Technologies, Huntsville, AL, USA). After overnight incubation, the cells were washed with Dulbecco’s phosphate-buffered saline (DPBS; Hyclone, Logan, UT, USA) to remove transfection complexes and further incubated in DMEM containing 2% FBS. The supernatants were collected after 48 and 72 h, and SARS-CoV-2pps were concentrated using PEG-8000 (Sigma-Aldrich) and resuspended in DPBS for storage. The viral stock titers were determined using the Luciferase Assay System (Promega, Madison, WI, USA), as previously described [22].

### 2.3. Drug Candidate Preparation

The medicinal herb candidates evaluated in this study were water, methanol, and ethanol extracts prepared from referenced medicinal plant materials [23]. The plant materials were obtained from the Hung Chuan Chinese Medicine Store (Kaohsiung City, Taiwan) and authenticated through anatomical examination and high-performance liquid chromatography (HPLC) analysis. The reference materials were deposited in the Kaohsiung Medical University Herbarium. Commercial herbal combination formulas were supplied by KO DA Pharmaceutical Co., Ltd. (Taoyuan, Taiwan), with individual components listed in their standard preparation (https://www.koda.com.tw/pro01d_e.aspx?type=02; accessed on 18 June 2025). All materials were extracted using standard protocols for hot-water extraction [24], methanol extraction [25], and ethanol extraction [26]. The water-soluble extracts were reconstituted in ddH_2_O, while methanol and ethanol extracts were reconstituted in dimethyl sulfoxide (DMSO; Sigma-Aldrich). Pure compounds were obtained from Sigma-Aldrich and dissolved in DMSO. For all assays, the drugs were diluted in culture media to their final working concentrations, ensuring that the DMSO content was less than 0.5%.

### 2.4. Cytotoxicity Assay

Huh-7 cells were seeded in 96-well plates (1 × 10^4^ cells per well) and treated with various concentrations of drug candidates for 72 h at 37 °C. After treatment, cell viability was assessed using the Cell Counting Kit-8 (CCK-8; Sigma-Aldrich) according to the manufacturer’s instructions. Following the addition of the CCK-8 reagent, cells were incubated at 37 °C for 2 h, and the optical density (OD) was measured at 450 nm using a microplate reader. Cytotoxicity curves and 50% cytotoxic concentrations (CC_50_) for each extract were determined using non-linear regression analysis in the GraphPad Prism software (Version 9.0.2). Screening concentrations were selected based on predicted values yielding ≥90% cell viability.

### 2.5. Entry Inhibition Assay

Huh-7 cells were seeded in 96-well plates (1 × 10^4^ cells per well) and inoculated with virus–drug mixtures containing SARS-CoV-2pps (MOI = 0.01) and each drug candidate at the respective screening concentration for 2 h at 37 °C. After infection, the cells were washed with DPBS and further incubated in DMEM supplemented with 2% FBS for 72 h. Cell lysates were collected, and viral infectivity was determined using the Luciferase Assay System (Promega) following the manufacturer’s protocol. Test candidates that showed statistical significance and reduced the SARS-CoV-2pp luciferase reporter signal to below 10,000 relative light units (RLU) were considered effective.

### 2.6. Inactivation Assay

Caffeic acid (100 μM) and SARS-CoV-2pps were mixed in an Eppendorf tube and incubated at 37 °C for 1 h. After the incubation, the virus–drug mixture was diluted 20-fold and added to Huh-7 cells seeded in 96-well plates (1 × 10^4^ cells per well). The final MOI of SARS-CoV-2pps was 0.01. After infection for 2 h at 37 °C, the cells were washed with DPBS and further incubated in DMEM supplemented with 2% FBS for 72 h. Cell lysates were collected, and viral infectivity was determined using the Luciferase Assay System (Promega) following the manufacturer’s protocol.

### 2.7. Pretreatment Assay

Huh-7 cells were seeded in 96-well plates (1 × 10^4^ cells per well) and incubated with caffeic acid (100 μM) for 24 h. After the incubation, supernatants containing the drug were removed, and the cells were infected with SARS-CoV-2pps (MOI = 0.01) for 2 h at 37 °C. After infection, the cells were washed with DPBS and further incubated in DMEM supplemented with 2% FBS for 72 h. Cell lysates were collected, and viral infectivity was determined using the Luciferase Assay System (Promega) following the manufacturer’s protocol.

### 2.8. Statistical Analysis

Statistical analysis was conducted using GraphPad Prism 9.0.2 (GraphPad Software, San Diego, CA, USA). Data are presented as mean ± standard deviation (SD) from three independent experiments. Statistical significance was determined using one-way ANOVA followed by Dunnett’s multiple comparisons test (*p* < 0.05).

## 3. Results

### 3.1. Screening of Medicinal Herbal Extracts and Formulas for Viral Entry Inhibition

**Figure 1:**
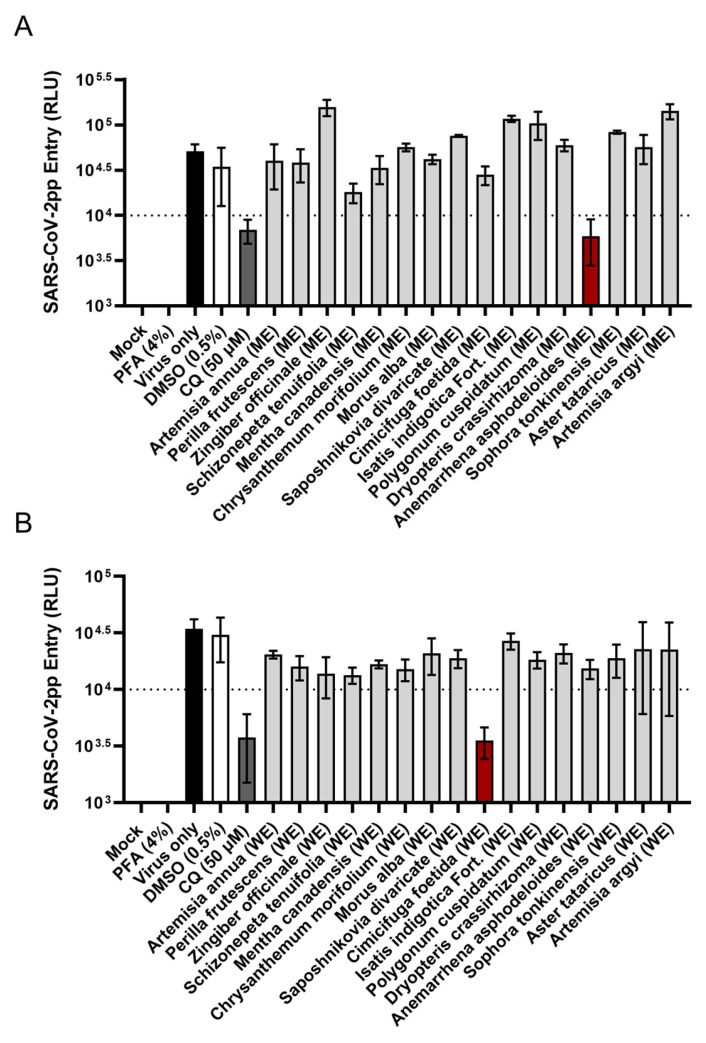
Inhibition of SARS-CoV-2pp entry by the extracts of heat-clearing and detoxifying medicinal herbs. (**A**) Methanol extracts (MEs) and (**B**) water extracts (WEs) of each herb were examined. SARS-CoV-2pp entry was quantified by luciferase reporter activity, expressed as relative light units (RLU). Extracts that reduced the luciferase signal to below 10,000 RLU were considered effective (red bar). Chloroquine (CQ) was used as a positive control (dark grey bar). Cells treated with 0.5% DMSO served as a solvent negative control (white bar), while cells fixed with paraformaldehyde (PFA) before infection served as a non-entry negative control. Data shown are mean ± SD from three independent experiments.

**Figure 2:**
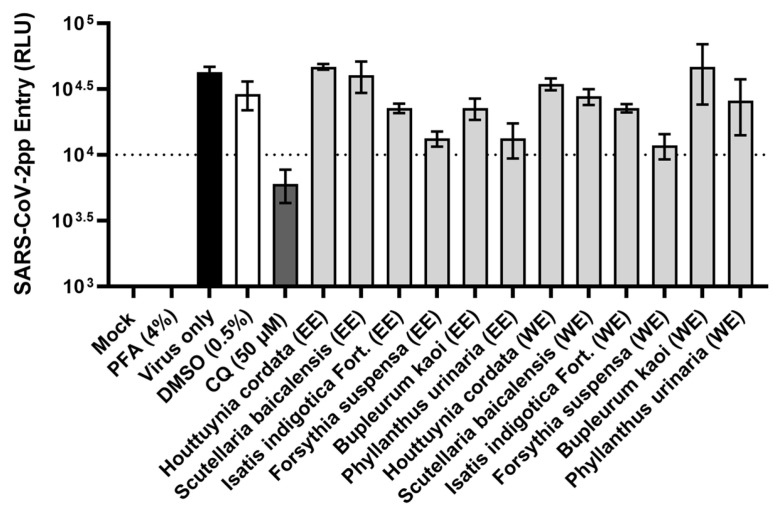
Inhibition of SARS-CoV-2pp entry by the ethanol extracts (EEs) and water extracts (WEs) of antiviral medicinal herbs. SARS-CoV-2pp entry was quantified by luciferase reporter activity, expressed as relative light units (RLU). Extracts that reduced the luciferase signal to below 10,000 RLU were considered effective. Chloroquine (CQ) was used as a positive control (dark grey bar). Cells treated with 0.5% DMSO served as a solvent negative control (white bar), while cells fixed with paraformaldehyde (PFA) before infection served as a non-entry negative control. Data shown are mean ± SD from three independent experiments.

**Figure 3:**
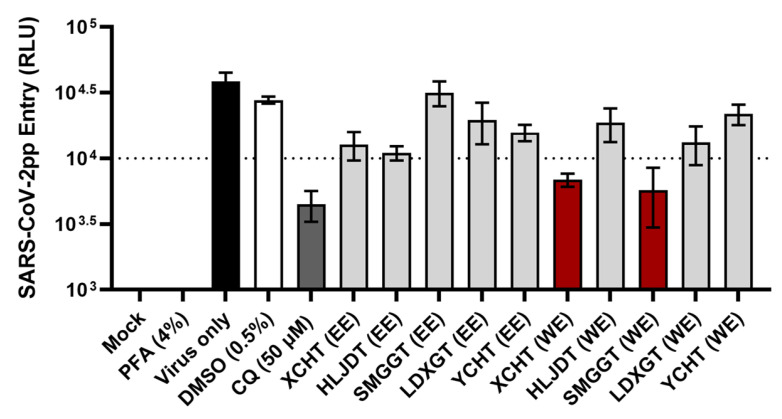
Inhibition of SARS-CoV-2pp entry by the ethanol extracts (EEs) and water extracts (WEs) of herbal combination formulas. SARS-CoV-2pp entry was quantified by luciferase reporter activity, expressed as relative light units (RLU). Extracts that reduced the luciferase signal to below 10,000 RLU were considered effective (red bars). Chloroquine (CQ) was used as a positive control (dark grey bar). Cells treated with 0.5% DMSO served as a solvent negative control (white bar), while cells fixed with paraformaldehyde (PFA) before infection served as a non-entry negative control. Data shown are mean ± SD from three independent experiments.

Alcoholic extracts and water extracts (WEs) were prepared from three panels of traditional herbal medicines. The first panel (Table 1 and Figure 1) consists of methanol extracts (MEs) and WEs from medicinal herbs that are known for their heat-clearing and detoxifying properties (*Artemisia annua*, *Isatis indigotica* Fort., *Dryopteris crassirhizoma*, *Anemarrhena asphodeloides*, *Sophora tonkinensis*), exterior-releasing effect (*Perilla frutescens*, *Schizonepeta tenuifolia*, *Mentha canadensis*, *Chrysanthemum morifolium*, *Morus alba*, *Saposhnikovia divaricate*, and *Cimicifuga foetida*), interior-warming effect (*Zingiber officinale*), phlegm-dispelling effect (*Aster tataricus*), and blood-regulating effect (*Polygonum cuspidatum*, *Artemisia argyi*) [23], which are often used for treating febrile diseases and infections. The second panel (Table 2 and Figure 2) consists of ethanol extracts (EEs) and WEs from medicinal herbs with documented antiviral activities [27]. *Houttuynia cordata*, *Scutellaria baicalensis*, *Isatis indigotica* Fort., and *Forsythia suspensa* also have heat-clearing effects [23] and have previously demonstrated in vitro antiviral activity against severe acute respiratory syndrome coronavirus (SARS-CoV) [28,29,30]. These herbs are among the components in the traditional Chinese medicine formulas used for COVID-19 treatment, such as NRICM101 [31], Shuanghuanglian [32], and Lianhuaqingwen [33]. *Phyllanthus urinaria* and *Bupleurum kaoi*, on the other hand, have demonstrated anti-hepatitis C virus (HCV) activity [34,35]. The third panel (Table 3 and Figure 3) consists of EEs and WEs from the heat-clearing herbal combination formulas Xiao Chai Hu Tang (XCHT), Huang Lian Jie Du Tang (HLJDT), Sheng Ma Ge Gen Tang (SMGGT), Long Dan Xie Gan Tang (LDXGT), and Yin Chen Hao Tang (YCHT) [23]. The cytotoxicity profiles for each extract were characterized to determine the 50% cytotoxic concentration (CC_50_) and the screening concentration (SC) that maintained at least 90% cell viability (Table 1, Table 2 and Table 3).

To assess the SARS-CoV-2 entry inhibitory potential of these medicinal herbs and combination formulas, a SARS-CoV-2 pseudoparticle (SARS-CoV-2pp) system that we previously established [22] was used for the inhibition assay. To validate the inhibition of viral entry, chloroquine, a known SARS-CoV-2 entry inhibitor that prevents endosomal acidification [36], was included as a positive control. Cells treated with 0.5% DMSO were used as a solvent negative control, and cells fixed with 4% paraformaldehyde (PFA) prior to SARS-CoV-2pp infection served as a non-entry negative control to account for background signal. The screening results indicated that methanol extract of *Anemarrhena asphodeloides* rhizome (Figure 1A), water extract of *Cimicifuga foetida* rhizome (Figure 1B), and water extracts of XCHT and SMGGT (Figure 3) were the most effective in blocking SARS-CoV-2pp entry, reducing the pseudoparticles’ luciferase reporter signal to below 10,000 relative light units (RLU) and similar to those of chloroquine. The percentages of inhibition were 88%, 90%, 82%, and 85%, respectively, compared to the virus-only group.

### 3.2. Dose-Dependent Antiviral Activity of Cimicifuga foetida Rhizome Water Extract

**Figure 4:**
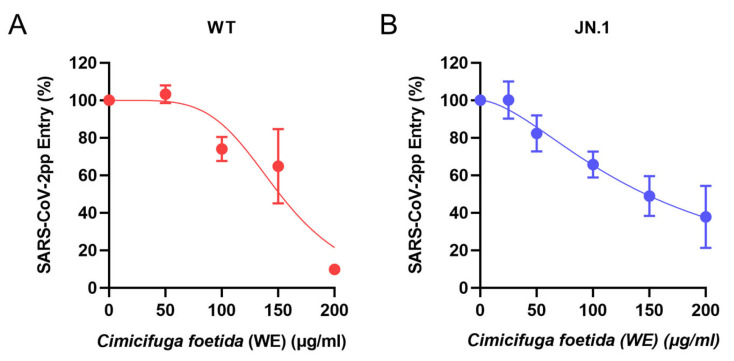
Dose-dependent inhibition of SARS-CoV-2pp entry by *Cimicifuga foetida* rhizome water extract. Entry of SARS-CoV-2pps bearing the (**A**) wild-type (WT) or (**B**) JN.1 spike was quantified by luciferase reporter activity and normalized to the drug = 0 μg/mL group. Data shown are mean ± SD from three independent experiments. A least-squares-fit non-linear regression model was used to predict the 50% effective concentration (EC_50_).

Given that the water extract of *Cimicifuga foetida* rhizome was the most effective in blocking SARS-CoV-2pp entry and that the herb is also an ingredient in the SMGGT formula (*Puerariae Radix*, *Cimicifugae Rhizoma*, *Paeoniae Alba Radix*, *Glycynhizae Radix et Rhizoma*, *Zingiberis Rhizoma Recens*) [23], we further explored the antiviral efficacy of the extract. The *Cimicifuga foetida* rhizome water extract showed dose-dependent inhibition of SARS-CoV-2pp entry (Figure 4A), yielding an estimated 50% effective concentration (EC_50_) of 151.5 μg/mL. We also examined the antiviral dose response on the currently dominant lineage JN.1 prototype and found that *Cimicifuga foetida* rhizome water extract also inhibited JN.1 pseudoparticle entry with an EC_50_ of 147.8 μg/mL (Figure 4B). These results confirm the inhibitory activity of the extract on SARS-CoV-2pp entry and highlight the extract’s potential value towards the newly emerging variants of JN.1 sublineages.

### 3.3. Characterization and Identification of Bioactive Antiviral Compounds

**Figure 5:**
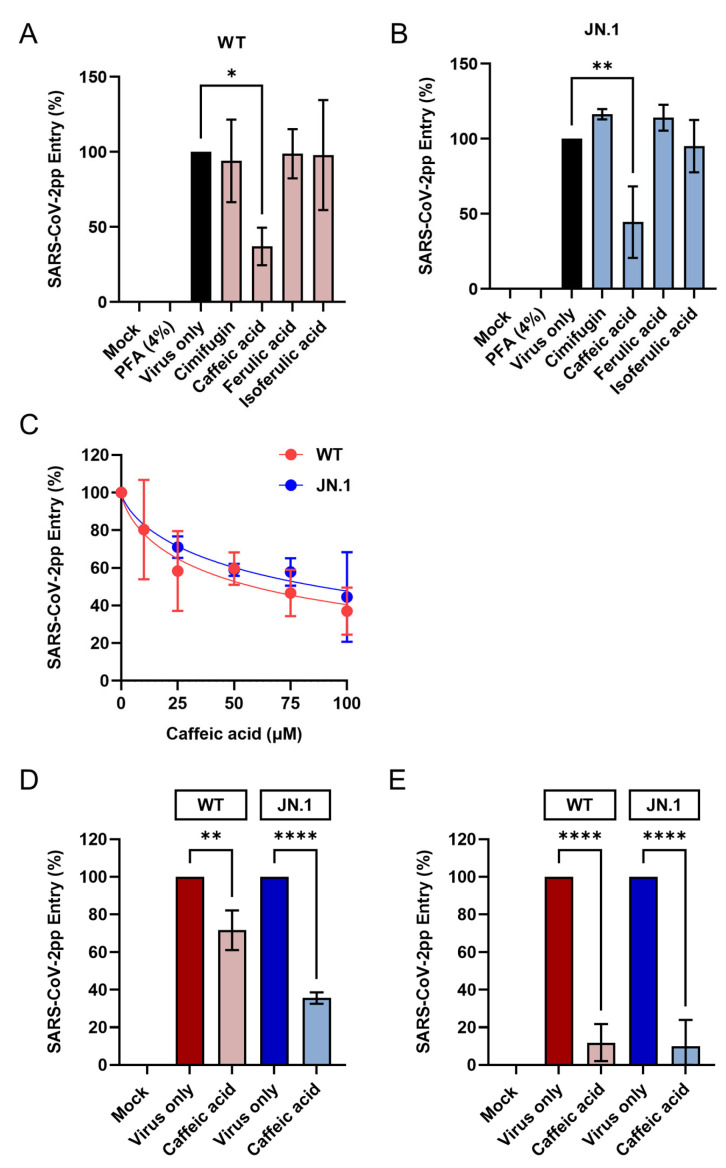
Mechanistic evaluation of the antiviral activity of *Cimicifuga foetida* rhizome water extract. (**A**,**B**) Inhibitory effects of the major compounds (100 μM) on entry of (**A**) WT and (**B**) JN.1 variant SARS-CoV-2pp entry. (**C**) Dose–response curve of caffeic acid against SARS-CoV-2pp entry. (**D**) Virucidal activity of caffeic acid (100 μM) assessed by inactivation assay. (**E**) Inhibition of SARS-CoV-2pp entry following pretreatment of host cells with caffeic acid (100 μM). Statistical significance was determined using one-way ANOVA followed by Dunnett’s multiple comparisons test (* *p* < 0.05; ** *p* < 0.01; **** *p* < 0.0001).

To identify the antiviral mechanism of *Cimicifuga foetida* rhizome water extract, we next investigated its major components, including isoferulic acid, ferulic acid, caffeic acid, and cimifugin [23,37,38]. The cytotoxicity profiles of the compounds were first determined (Table 4), and 100 μM (a non-cytotoxic concentration) was used for the entry-inhibition assay. The results indicated that, out of the four candidates, only caffeic acid blocked both WT and JN.1 SARS-CoV-2pp entry at 100 μM (Figure 5A,B) and demonstrated dose-dependent inhibition (Figure 5C). The estimated EC_50_’s were 58.62 μM and 88.32 μM for the WT and JN.1 strains, respectively. Additionally, caffeic acid was able to inactivate the pseudoparticles directly (Figure 5D) and also inhibit pseudoparticle entry following 24 h pretreatment of host cells (Figure 5E). These findings suggest that the compound exhibits both virucidal activity and the capacity to interact with host-cell factors involved in viral entry.

## 4. Discussion

Viral entry is the first step in viral infection; therefore, entry inhibitors represent a promising class of antivirals [39] that could not only provide prophylactic effect but also reduce secondary infections from the infected cells. In the current study, we identified several herbal candidates, including extracts of *Anemarrhena asphodeloides*, *Cimicifuga foetida*, XCHT, and SMGGT, as potential sources for the development of SARS-CoV-2-specific entry inhibitors. We have previously shown that several herbal extract candidates on our list demonstrated inhibitory effects on viral entry of other viruses. Specifically, the methanol extract of *Perilla frutescens* blocked viral attachment and neutralized pseudoparticles of Ebola virus (EBOV) [40]; the methanol extract of *Polygonum cuspidatum* was shown to inactivate dengue virus (DENV) and inhibit viral attachment and entry/fusion events [41]; the acetone extract of *Phyllanthus urinaria* inhibited the attachment and free viral particles of hepatitis C virus (HCV) [34]; whereas the methanol extract of *Bupleurum kaoi* mainly targeted the fusion step of HCV entry [35]. These extracts, however, did not inhibit SARS-CoV-2pp entry in the current study, suggesting that the reported antiviral activities are virus-specific. In addition, a mechanistic study on the formula NRICM101 suggested that *Scutellaria baicalensis* was able to neutralize SARS-CoV-2 in a plaque reduction neutralization test [31], yet we did not observe its effect on entry in our study. This discrepancy could potentially be explained by the different cell types (which may provide different entry routes for SARS-CoV-2) and assay conditions used. Of note, while our study employs a mechanism-specific approach to identify entry inhibitors, we cannot rule out whether the medicinal herbs and formulas could potentially harbor antiviral effects against SARS-CoV-2 through other mechanisms, including inhibitory effects on other stages of the viral replicative cycle and immunomodulation.

One of the identified candidate herbs, *Anemarrhena asphodeloides*, is a plant in the asparagus family. The rhizome of the herb is used in traditional Chinese medicine and contains key bioactive components, including timosaponin B-II (≥3.0%), mangiferin (≥0.7%), neomangiferin, timosaponin A-III, and isomangiferin [23,42,43,44]. Extracts from *Anemarrhena asphodeloides* and its major components have also been reported for their antiviral activities. Timosaponin B-II has shown anti-enterovirus 71 (EV71) effects [45]. Timosaponin A-III [46] and three additional compounds, (−)-(R)-nyasol, (−)-(R)-4′-O-methylnyasol, and broussonin A, isolated from the methanol extract of *Anemarrhena asphodeloides* rhizome [47] were able to inhibit respiratory syncytial virus (RSV) infection. In addition, other classes of saponins have been suggested to prevent SARS-CoV-2 entry by disrupting the viral envelope or interfering with the spike-ACE2 interaction [48]. Given the high percentage of timosaponins in *Anemarrhena asphodeloides*, it is plausible that these compounds may exert a similar effect on SARS-CoV-2 entry. Mangiferin, another major component of *Anemarrhena asphodeloides*, has demonstrated antiviral activities against herpes simplex virus type 1 (HSV-1) [49,50] and human immunodeficiency virus type 1 (HIV-1) [51]. Our finding that the methanol extract of *Anemarrhena asphodeloides* rhizome inhibits SARS-CoV-2pp entry warrants further investigation to identify the molecular mechanisms in targeting viral entry steps and active antiviral compounds.

The two combination candidates identified, XCHT (also known as Shosaikoto in Kampo medicine or Minor Bupleurum Combination) and SMGGT (also known as Shomakakkonto or Cimicifuga and Pueraria Combination), are classical herbal combination formulas conventionally used for treating febrile diseases, such as those from viral infections. XCHT has been documented for its immunomodulatory, anti-inflammatory, and anti-oxidant activities [52], and antiviral effects against hepatitis B virus (HBV) [53] and Coxsackie B virus type 1 (CVB1) [54]. For SMGGT, antiviral activities were reported for measles virus [55], EV71 [56], and RSV [57]. Our study is the first to demonstrate the formulas’ effects on preventing SARS-CoV-2pp entry. XCHT typically contains *Bupleuri Radix*, *Scutellariae Radix*, *Ginseng Radix*, *Glycyrrhizae Radix et Rhizoma Praeparatum cum Melle*, *Pinelliae Rhizoma Praeparatum*, *Zingiberis Rhizoma Recens*, and *Jujubae Fructus*; whereas SMGGT typically contains *Puerariae Radix*, *Cimicifugae Rhizoma*, *Paeoniae Alba Radix*, *Glycynhizae Radix et Rhizoma*, *Zingiberis*, and *Rhizoma Recens* [23]. Among the ingredients, the major components of *Ginseng* and *Glycyrrhizae* were reported to have direct antiviral effects on virus particles, entry, and replication of various viruses [58,59,60]. Gallic acid, methyl gallate, and pentagalloylglucose isolated from *Paeoniae Alba Radix* inhibit influenza A virus replication and reduce neuraminidase activity [61]. The water extract of fresh ginger (*Zingiberis Rhizoma Recens*), but not dried ginger, was shown to inhibit RSV entry by reducing viral attachment [62]. Interestingly, a recent study reported that the water extract of *Scutellaria baicalensis* inhibits SARS-CoV-2pp entry at higher concentrations (250 μg/mL) [63], but we did not observe this effect at the lower concentration used in our study (100 μg/mL). The active components responsible for SARS-CoV-2 entry inhibition and the potential of these two formulas to suppress other stages of the SARS-CoV-2 viral life cycle require further investigation.

The most potent candidate identified in our study is *Cimicifuga foetida* water extract. The herb and its major components have exhibited antiviral effects on various viruses. Water extracts of the herb have been shown to inhibit RSV infection and viral attachment dose-dependently [64], and cimicifugin was found to inhibit RSV attachment and internalization [65]. Another major compound, ferulic acid, possesses multiple antiviral mechanisms against both viruses and hosts [66]. Its derivatives have been shown to block replication of the coronaviruses HCoV-229E and SARS-CoV-2 [67,68]. Virtual screening studies predicted that ferulic acid could interact with SARS-CoV-2 viral proteins including spike [69] and membrane [70] proteins, which are crucial for viral attachment and virion assembly, respectively. Caffeic acid, on the other hand, demonstrated antiviral activities against influenza A virus [71], HCV [72], thrombocytopenia syndrome virus [73], and Ilhéus virus [74]. Notably, caffeic acid was shown to inhibit the replication cycle and viral attachment of HCoV-NL63 [75], a commonly used surrogate virus for SARS-CoV-2 [76].

In this study, we identified caffeic acid as a key bioactive compound in the *Cimicifuga foetida* rhizome extract that inhibits SARS-CoV-2pp entry. Mechanistic assays demonstrated that caffeic acid could both directly inactivate pseudoparticles and reduce viral entry when used to pretreat host cells, suggesting dual antiviral activity. These findings are supported by recent molecular docking studies, which predict that caffeic acid can bind not only to the SARS-CoV-2 spike protein [77,78], but also to host entry factors such as ACE2 and HSPA5 [78,79]. Specifically, caffeic acid binds with the spike receptor-binding domain (RBD) at residues Leu441, Tyr495, and Phe497 (binding energy = −6.43 kcal/mol, inhibition constant (Ki) = 19.36 μM); ACE2 at residues Leu73, Ala99, Leu100, Lys74, and Asn103 (binding energy = −5.31 kcal/mol, Ki = 127.93 μM); and HSPA5 substrate-binding domain β (SBDβ, which recognizes SARS-CoV-2 spike) at residues Phe451, Val453, Ile483(2), Leu480, and Ile493 (binding energy = −6.2 kcal/mol) [78,79]. Whether these are the specific target(s) of caffeic acid remains to be validated. In addition, some reports suggest that cathepsin B may be involved in the endosomal entry route of SARS-CoV-2 [80], and caffeic acid has been shown to inhibit cathepsin B activity with an IC_50_ of 110 ± 10 μM [81]. These studies are consistent with our experimental data, collectively indicating that caffeic acid may interfere with SARS-CoV-2 entry through multiple mechanisms, including direct viral targeting and modulation of host factors. To confirm its antiviral efficacy under physiological conditions, future studies should assess caffeic acid’s activity against infectious SARS-CoV-2 strains in vitro and in vivo. Further investigation of its pharmacokinetics, bioavailability, efficacy, and safety will also be essential for evaluating its therapeutic potential.

## 5. Conclusions

This study highlights the potential of the extracts from *Anemarrhena asphodeloides*, *Cimicifuga foetida*, XCHT, and SMGGT as effective inhibitors of SARS-CoV-2 entry. Furthermore, caffeic acid was identified as a bioactive compound from *Cimicifuga foetida* rhizome. Our findings support further investigation and development of these medicinal herbs, herbal formulas, and compound as candidates for COVID-19 prevention and promising sources for developing novel entry inhibitors against SARS-CoV-2.

## Figures and Tables

**Table 1:** List of heat-clearing and detoxifying medicinal herb candidates.

Species	Part (s)	CC_50_ (μg/mL)		SC (μg/mL)	
		ME	WE	ME	WE
*Artemisia annua*	*Herba*	284.2	>800	100	5
*Perilla frutescens*	*Folium*	167.9	>800	125	200
*Zingiber officinale*	*Rhizoma* (dried)	51.75	>800	48	20
*Schizonepeta tenuifolia*	*Herba*	727.6	>800	200	50
*Mentha canadensis*	*Herba*	330	>800	160	200
*Chrysanthemum morifolium*	*Flos*	1082	>800	200	100
*Morus alba*	*Folium*	505	>800	200	100
*Saposhnikovia divaricate*	*Radix*	972	>800	200	200
*Cimicifuga foetida*	*Rhizoma*	46.17	>800	20	200
*Isatis indigotica* Fort.	*Folium*	624.3	>800	200	200
*Polygonum cuspidatum*	*Radix*	133.8	617.9	40	200
*Dryopteris crassirhizoma*	*Rhizoma*	1723	>800	200	200
*Anemarrhena asphodeloides*	*Rhizoma*	766.6	>800	200	200
*Sophora tonkinensis*	*Radix*	402.5	>800	50	30
*Aster tataricus*	*Radix et rhizoma*	882.2	>800	200	200
*Artemisia argyi*	*Folium*	208.2	595.6	50	250

CC_50_, 50% cytotoxic concentration; SC, screening concentration; ME, methanol extract; WE, water extract.

**Table 2:** List of antiviral medicinal herb candidates.

Species	Part (s)	CC_50_ (μg/mL)		SC (μg/mL)	
		EE	WE	EE	WE
*Houttuynia cordata*	*Herba*	>20	>100	20	100
*Scutellaria baicalensis*	*Radix*	>7.8	>100	1.5	100
*Isatis indigotica* Fort.	*Radix*	>125	>125	10	5
*Forsythia suspensa*	*Fructus*	109.7	>125	10	50
*Bupleurum kaoi*	*Radix*	80.92	>8	40	8
*Phyllanthus urinaria*	*Herba*	>7.8	>125	5	40

CC_50_, 50% cytotoxic concentration; SC, screening concentration; EE, ethanol extract; WE, water extract.

**Table 3:** List of herbal combination formula candidates.

Formula	CC_50_ (μg/mL)		SC (μg/mL)	
	EE	WE	EE	WE
Xiao Chai Hu Tang (XCHT; Minor Bupleurum Combination)	233	>125	100	80
Huang Lian Jie Du Tang (HLJDT; Coptis & Scute Combination)	185	>125	50	50
Sheng Ma Ge Gen Tang (SMGGT; Cimicifuga & Pueraria Combination)	154.9	>125	80	45
Long Dan Xie Gan Tang (LDXGT; Gentiana Combination)	>250	>125	200	40
Yin Chen Hao Tang (YCHT; Capillaris Combination)	104.3	>125	20	30

CC_50_, 50% cytotoxic concentration; SC, screening concentration; EE, ethanol extract; WE, water extract.

**Table 4:** Cytotoxicity profile of major compounds from *Cimicifuga foetida* rhizome water extract.

Compound	CC_50_ (μM)
Cimifugin	4206
Caffeic acid	882.8
Ferulic acid	4843
Iosferulic acid	6710

## Data Availability

Data is contained within the article.

## References

[1] World Health Organization Coronavirus Disease (COVID-19) Epidemiological Updates and Monthly Operational Updates. https://www.who.int/emergencies/diseases/novel-coronavirus-2019/situation-reports.

[2] Moghadas S.M., Vilches T.N., Zhang K., Wells C.R., Shoukat A., Singer B.H., Meyers L.A., Neuzil K.M., Langley J.M., Fitzpatrick M.C. (2021). The impact of vaccination on COVID-19 outbreaks in the United States. Clin. Infect. Dis..

[3] Carabelli A.M., Peacock T.P., Thorne L.G., Harvey W.T., Hughes J., Peacock S.J., Barclay W.S., de Silva T.I., Towers G.J., Genomics UK Consortium (2023). SARS-CoV-2 variant biology: Immune escape, transmission and fitness. Nat. Rev. Microbiol..

[4] Wu N., Joyal-Desmarais K., Ribeiro P.A.B., Vieira A.M., Stojanovic J., Sanuade C., Yip D., Bacon S.L. (2023). Long-term effectiveness of COVID-19 vaccines against infections, hospitalisations, and mortality in adults: Findings from a rapid living systematic evidence synthesis and meta-analysis up to December, 2022. Lancet Respir. Med..

[5] Lau J.J., Cheng S.M.S., Leung K., Lee C.K., Hachim A., Tsang L.C.H., Yam K.W.H., Chaothai S., Kwan K.K.H., Chai Z.Y.H. (2023). Real-world COVID-19 vaccine effectiveness against the Omicron BA.2 variant in a SARS-CoV-2 infection-naive population. Nat. Med..

[6] U.S. Food and Drug Administration FDA Considerations and Recommendations for the 2025–2026 Formula of COVID-19 Vaccines in the United States. https://www.fda.gov/media/186594/download.

[7] U.S. Food and Drug Administration COVID-19 Vaccines (2025–2026 Formula) for Use in the United States Beginning in Fall 2025. https://www.fda.gov/vaccines-blood-biologics/industry-biologics/covid-19-vaccines-2025-2026-formula-use-united-states-beginning-fall-2025.

[8] U.S. Food and Drug Administration Coronavirus (COVID-19)|Drugs. https://www.fda.gov/drugs/emergency-preparedness-drugs/coronavirus-covid-19-drugs.

[9] Infectious Diseases Society of America (IDSA) IDSA Guidelines on the Treatment and Management of Patients with COVID-19. https://www.idsociety.org/COVID19guidelines.

[10] Hoffmann M., Kleine-Weber H., Schroeder S., Kruger N., Herrler T., Erichsen S., Schiergens T.S., Herrler G., Wu N.H., Nitsche A. (2020). SARS-CoV-2 Cell Entry Depends on ACE2 and TMPRSS2 and Is Blocked by a Clinically Proven Protease Inhibitor. Cell.

[11] Cantuti-Castelvetri L., Ojha R., Pedro L.D., Djannatian M., Franz J., Kuivanen S., van der Meer F., Kallio K., Kaya T., Anastasina M. (2020). Neuropilin-1 facilitates SARS-CoV-2 cell entry and infectivity. Science.

[12] Wang K., Chen W., Zhang Z., Deng Y., Lian J.Q., Du P., Wei D., Zhang Y., Sun X.X., Gong L. (2020). CD147-spike protein is a novel route for SARS-CoV-2 infection to host cells. Signal Transduct. Target. Ther..

[13] Carlos A.J., Ha D.P., Yeh D.W., Van Krieken R., Tseng C.C., Zhang P., Gill P., Machida K., Lee A.S. (2021). The chaperone GRP78 is a host auxiliary factor for SARS-CoV-2 and GRP78 depleting antibody blocks viral entry and infection. J. Biol. Chem..

[14] Shang J., Wan Y., Luo C., Ye G., Geng Q., Auerbach A., Li F. (2020). Cell entry mechanisms of SARS-CoV-2. Proc. Natl. Acad. Sci. USA.

[15] Bayati A., Kumar R., Francis V., McPherson P.S. (2021). SARS-CoV-2 infects cells after viral entry via clathrin-mediated endocytosis. J. Biol. Chem..

[16] Zhao M.M., Yang W.L., Yang F.Y., Zhang L., Huang W.J., Hou W., Fan C.F., Jin R.H., Feng Y.M., Wang Y.C. (2021). Cathepsin L plays a key role in SARS-CoV-2 infection in humans and humanized mice and is a promising target for new drug development. Signal Transduct. Target. Ther..

[17] V’Kovski P., Kratzel A., Steiner S., Stalder H., Thiel V. (2021). Coronavirus biology and replication: Implications for SARS-CoV-2. Nat. Rev. Microbiol..

[18] Takayama S., Namiki T., Odaguchi H., Arita R., Hisanaga A., Mitani K., Ito T. (2021). Prevention and Recovery of COVID-19 Patients with Kampo Medicine: Review of Case Reports and Ongoing Clinical Trials. Front. Pharmacol..

[19] Al-Kuraishy H.M., Al-Fakhrany O.M., Elekhnawy E., Al-Gareeb A.I., Alorabi M., De Waard M., Albogami S.M., Batiha G.E. (2022). Traditional herbs against COVID-19: Back to old weapons to combat the new pandemic. Eur. J. Med. Res..

[20] Panyod S., Ho C.T., Sheen L.Y. (2020). Dietary therapy and herbal medicine for COVID-19 prevention: A review and perspective. J. Tradit. Complement. Med..

[21] Al-Jamal H., Idriss S., Roufayel R., Abi Khattar Z., Fajloun Z., Sabatier J.M. (2024). Treating COVID-19 with Medicinal Plants: Is It Even Conceivable? A Comprehensive Review. Viruses.

[22] Belem W.F., Liu C.H., Hu Y.T., Burnouf T., Lin L.T. (2022). Validation of Viral Inactivation Protocols for Therapeutic Blood Products against Severe Acute Respiratory Syndrome Coronavirus-2 (SARS-CoV-2). Viruses.

[23] Taiwan Herbal Pharmacopeia 3rd Ed. Committee (2019). Taiwan Herbal Pharmacopeia.

[24] Hsu W.C., Chang S.P., Lin L.C., Li C.L., Richardson C.D., Lin C.C., Lin L.T. (2015). *Limonium sinense* and gallic acid suppress hepatitis C virus infection by blocking early viral entry. Antiviral Res..

[25] Hung T.C., Jassey A., Lin C.J., Liu C.H., Lin C.C., Yen M.H., Lin L.T. (2018). Methanolic Extract of *Rhizoma coptidis* Inhibits the Early Viral Entry Steps of Hepatitis C Virus Infection. Viruses.

[26] Yen F.L., Wu T.H., Lin L.T., Cham T.M., Lin C.C. (2008). Concordance between antioxidant activities and flavonol contents in different extracts and fractions of *Cuscuta chinensis*. Food Chem..

[27] Lin L.T., Hsu W.C., Lin C.C. (2014). Antiviral natural products and herbal medicines. J. Tradit. Complement. Med..

[28] Li S.Y., Chen C., Zhang H.Q., Guo H.Y., Wang H., Wang L., Zhang X., Hua S.N., Yu J., Xiao P.G. (2005). Identification of natural compounds with antiviral activities against SARS-associated coronavirus. Antivir. Res..

[29] Lin C.W., Tsai F.J., Tsai C.H., Lai C.C., Wan L., Ho T.Y., Hsieh C.C., Chao P.D. (2005). Anti-SARS coronavirus 3C-like protease effects of *Isatis indigotica* root and plant-derived phenolic compounds. Antivir. Res..

[30] Lau K.M., Lee K.M., Koon C.M., Cheung C.S., Lau C.P., Ho H.M., Lee M.Y., Au S.W., Cheng C.H., Lau C.B. (2008). Immunomodulatory and anti-SARS activities of *Houttuynia cordata*. J. Ethnopharmacol..

[31] Tsai K.C., Huang Y.C., Liaw C.C., Tsai C.I., Chiou C.T., Lin C.J., Wei W.C., Lin S.J., Tseng Y.H., Yeh K.M. (2021). A traditional Chinese medicine formula NRICM101 to target COVID-19 through multiple pathways: A bedside-to-bench study. Biomed. Pharmacother..

[32] Su H.X., Yao S., Zhao W.F., Li M.J., Liu J., Shang W.J., Xie H., Ke C.Q., Hu H.C., Gao M.N. (2020). Anti-SARS-CoV-2 activities in vitro of Shuanghuanglian preparations and bioactive ingredients. Acta Pharmacol. Sin..

[33] Li R., Hou Y., Huang J., Pan W., Ma Q., Shi Y., Li C., Zhao J., Jia Z., Jiang H. (2020). Lianhuaqingwen exerts anti-viral and anti-inflammatory activity against novel coronavirus (SARS-CoV-2). Pharmacol. Res..

[34] Chung C.Y., Liu C.H., Burnouf T., Wang G.H., Chang S.P., Jassey A., Tai C.J., Tai C.J., Huang C.J., Richardson C.D. (2016). Activity-based and fraction-guided analysis of *Phyllanthus urinaria* identifies loliolide as a potent inhibitor of hepatitis C virus entry. Antiviral. Res..

[35] Lin L.T., Chung C.Y., Hsu W.C., Chang S.P., Hung T.C., Shields J., Russell R.S., Lin C.C., Li C.F., Yen M.H. (2015). Saikosaponin b2 is a naturally occurring terpenoid that efficiently inhibits hepatitis C virus entry. J. Hepatol..

[36] Wang M., Cao R., Zhang L., Yang X., Liu J., Xu M., Shi Z., Hu Z., Zhong W., Xiao G. (2020). Remdesivir and chloroquine effectively inhibit the recently emerged novel coronavirus (2019-nCoV) in vitro. Cell Res..

[37] Mao H., Zhang Y., Chen G. (2019). Determination of three phenolic acids in *Cimicifugae rhizoma* by capillary electrophoresis with a graphene–phenolic resin composite electrode. Anal. Methods.

[38] Guo Y., Yin T., Wang X., Zhang F., Pan G., Lv H., Wang X., Owoicho Orgah J., Zhu Y., Wu H. (2017). Traditional uses, phytochemistry, pharmacology and toxicology of the genus *Cimicifuga*: A review. J. Ethnopharmacol..

[39] Pattnaik G.P., Chakraborty H. (2020). Entry Inhibitors: Efficient Means to Block Viral Infection. J. Membr. Biol..

[40] Kuo Y.T., Liu C.H., Corona A., Fanunza E., Tramontano E., Lin L.T. (2021). The Methanolic Extract of *Perilla frutescens* Robustly Restricts Ebola Virus Glycoprotein-Mediated Entry. Viruses.

[41] Kuo Y.T., Liu C.H., Li J.W., Lin C.J., Jassey A., Wu H.N., Perng G.C., Yen M.H., Lin L.T. (2020). Identification of the phytobioactive *Polygonum cuspidatum* as an antiviral source for restricting dengue virus entry. Sci. Rep..

[42] Sun Y., Du Y., Liu Y., Chang L., Ren Y., Cao L., Sun Q., Shi X., Wang Q., Zhang L. (2012). Simultaneous determination of nine components in *Anemarrhena asphodeloides* by liquid chromatography-tandem mass spectrometry combined with chemometric techniques. J. Sep. Sci..

[43] Kim N., Ryu S.M., Lee D., Lee J.W., Seo E.K., Lee J.H., Lee D. (2014). A metabolomic approach to determine the geographical origins of *Anemarrhena asphodeloides* by using UPLC-QTOF MS. J. Pharm. Biomed. Anal..

[44] Nian S.H., Li H.J., Liu E.H., Li P. (2017). Comparison of alpha-glucosidase inhibitory effect and bioactive constituents of Anemarrhenae Rhizoma and Fibrous Roots. J. Pharm. Biomed. Anal..

[45] Liu M., Tao L., Chau S.L., Wu R., Zhang H., Yang Y., Yang D., Bian Z., Lu A., Han Q. (2014). Folding fan mode counter-current chromatography offers fast blind screening for drug discovery. Case study: Finding anti-enterovirus 71 agents from *Anemarrhena asphodeloides*. J. Chromatogr. A.

[46] Youn U.J., Jang J.-E., Nam J.-W., Lee Y.J., Son Y.M., Shin H.J., Han A.-R., Chang J., Seo E.-K. (2011). Anti-respiratory syncytial virus (RSV) activity of timosaponin A-III from the rhizomes of *Anemarrhena asphodeloides*. J. Med. Plants Res..

[47] Bae G., Yu J.R., Lee J., Chang J., Seo E.K. (2007). Identification of nyasol and structurally related compounds as the active principles from *Anemarrhena asphodeloides* against respiratory syncytial virus (RSV). Chem. Biodivers..

[48] Mieres-Castro D., Mora-Poblete F. (2023). Saponins: Research Progress and Their Potential Role in the Post-COVID-19 Pandemic Era. Pharmaceutics.

[49] Wang W.D., Chen G. (2023). Antiviral activity of mangiferin from the rhizome of *Anemarrhena asphodeloides* against herpes simplex virus type 1. Asian Pac. J. Trop. Biomed..

[50] Zheng M.S., Lu Z.Y. (1990). Antiviral effect of mangiferin and isomangiferin on herpes simplex virus. Chin. Med. J..

[51] Wang R.R., Gao Y.D., Ma C.H., Zhang X.J., Huang C.G., Huang J.F., Zheng Y.T. (2011). Mangiferin, an anti-HIV-1 agent targeting protease and effective against resistant strains. Molecules.

[52] Arita R., Ono R., Saito N., Takayama S., Namiki T., Ito T., Ishii T. (2020). Kakkonto, shosaikoto, *Platycodon grandiflorum* root, and gypsum (a Japanese original combination drug known as saikatsugekito): Pharmacological review of its activity against viral infections and respiratory inflammatory conditions and a discussion of its applications to COVID-19. Tradit. Kampo Med..

[53] Chang J.S., Wang K.C., Liu H.W., Chen M.C., Chiang L.C., Lin C.C. (2007). Sho-saiko-to (Xiao-Chai-Hu-Tang) and crude saikosaponins inhibit hepatitis B virus in a stable HBV-producing cell line. Am. J. Chin. Med..

[54] Cheng P.W., Ng L.T., Lin C.C. (2006). Xiao chai hu tang inhibits CVB1 virus infection of CCFS-1 cells through the induction of Type I interferon expression. Int. Immunopharmacol..

[55] Huang S.P., Shieh G.J., Lee L., Teng H.J., Kao S.T., Lin J.G. (1997). Inhibition effect of shengma-gegen-tang on measles virus in Vero cells and human peripheral blood mononuclear cells. Am. J. Chin. Med..

[56] Chang J.S., Wang K.C., Chiang L.C. (2008). Sheng-Ma-Ge-Gen-Tang inhibited Enterovirus 71 infection in human foreskin fibroblast cell line. J. Ethnopharmacol..

[57] Wang K.C., Chang J.S., Chiang L.C., Lin C.C. (2011). Sheng-Ma-Ge-Gen-Tang (Shoma-kakkon-to) inhibited cytopathic effect of human respiratory syncytial virus in cell lines of human respiratory tract. J. Ethnopharmacol..

[58] Im K., Kim J., Min H. (2016). Ginseng, the natural effectual antiviral: Protective effects of Korean Red Ginseng against viral infection. J. Ginseng Res..

[59] Huo C., Baek J., Kim K.H. (2024). Antiviral potential of ginseng: Targeting human pathogenic viruses with compounds derived from ginseng. J. Ginseng Res..

[60] Huan C., Xu Y., Zhang W., Guo T., Pan H., Gao S. (2021). Research Progress on the Antiviral Activity of Glycyrrhizin and its Derivatives in Liquorice. Front. Pharmacol..

[61] Zhang T., Lo C.Y., Xiao M., Cheng L., Pun Mok C.K., Shaw P.C. (2020). Anti-influenza virus phytochemicals from Radix Paeoniae Alba and characterization of their neuraminidase inhibitory activities. J. Ethnopharmacol..

[62] Chang J.S., Wang K.C., Yeh C.F., Shieh D.E., Chiang L.C. (2013). Fresh ginger (*Zingiber officinale*) has anti-viral activity against human respiratory syncytial virus in human respiratory tract cell lines. J. Ethnopharmacol..

[63] Lin C.H., Chang H.J., Lin M.W., Yang X.R., Lee C.H., Lin C.S. (2024). Inhibitory Efficacy of Main Components of *Scutellaria baicalensis* on the Interaction between Spike Protein of SARS-CoV-2 and Human Angiotensin-Converting Enzyme II. Int. J. Mol. Sci..

[64] Wang K.C., Chang J.S., Chiang L.C., Lin C.C. (2012). *Cimicifuga foetida* L. Inhibited Human Respiratory Syncytial Virus in HEp-2 and A549 Cell Lines. Am. J. Chin. Med..

[65] Wang K.C., Chang J.S., Lin L.T., Chiang L.C., Lin C.C. (2012). Antiviral effect of cimicifugin from *Cimicifuga foetida* against human respiratory syncytial virus. Am. J. Chin. Med..

[66] Antonopoulou I., Sapountzaki E., Rova U., Christakopoulos P. (2021). Ferulic Acid From Plant Biomass: A Phytochemical with Promising Antiviral Properties. Front. Nutr..

[67] Pasquereau S., Galais M., Bellefroid M., Pachon Angona I., Morot-Bizot S., Ismaili L., Van Lint C., Herbein G. (2022). Ferulic acid derivatives block coronaviruses HCoV-229E and SARS-CoV-2 replication in vitro. Sci. Rep..

[68] Verzola M.M.S.A., de Almeida Marques D.P., da Silva E.B., Serafim M.S.M., Ferreira R.S., Fajtová P., Kohlhoff M., O’Donoghue A.J., Maltarollo V.G., Coelho-dos-Reis J.G.A. (2023). Synthesis of indole-based ferulic acid derivatives and in vitro evaluation of antiviral activity against SARS-CoV-2. Med. Chem. Res..

[69] Salman S., Shah F.H., Idrees J., Idrees F., Velagala S., Ali J., Khan A.A. (2020). Virtual Screening of Immunomodulatory Medicinal Compounds as Promising anti-SARS-CoV-2 Inhibitors. Future Virol..

[70] Bhowmik D., Nandi R., Jagadeesan R., Kumar N., Prakash A., Kumar D. (2020). Identification of potential inhibitors against SARS-CoV-2 by targeting proteins responsible for envelope formation and virion assembly using docking based virtual screening, and pharmacokinetics approaches. Infect. Genet. Evol..

[71] Utsunomiya H., Ichinose M., Ikeda K., Uozaki M., Morishita J., Kuwahara T., Koyama A.H., Yamasaki H. (2014). Inhibition by caffeic acid of the influenza A virus multiplication in vitro. Int. J. Mol. Med..

[72] Shirasago Y., Inamori Y., Suzuki T., Tanida I., Suzuki T., Sugiyama K., Wakita T., Hanada K., Fukasawa M. (2019). Inhibition Mechanisms of Hepatitis C Virus Infection by Caffeic Acid and Tannic Acid. Biol. Pharm. Bull..

[73] Ogawa M., Shirasago Y., Tanida I., Kakuta S., Uchiyama Y., Shimojima M., Hanada K., Saijo M., Fukasawa M. (2021). Structural basis of antiviral activity of caffeic acid against severe fever with thrombocytopenia syndrome virus. J. Infect. Chemother..

[74] Saivish M.V., Pacca C.C., da Costa V.G., de Lima Menezes G., da Silva R.A., Nebo L., da Silva G.C.D., de Aguiar Milhim B.H.G., da Silva Teixeira I., Henrique T. (2023). Caffeic Acid Has Antiviral Activity against Ilheus Virus In Vitro. Viruses.

[75] Weng J.R., Lin C.S., Lai H.C., Lin Y.P., Wang C.Y., Tsai Y.C., Wu K.C., Huang S.H., Lin C.W. (2019). Antiviral activity of Sambucus FormosanaNakai ethanol extract and related phenolic acid constituents against human coronavirus NL63. Virus Res..

[76] Castillo G., Mora-Diaz J.C., Breuer M., Singh P., Nelli R.K., Gimenez-Lirola L.G. (2023). Molecular mechanisms of human coronavirus NL63 infection and replication. Virus Res..

[77] Adem S., Eyupoglu V., Sarfraz I., Rasul A., Zahoor A.F., Ali M., Abdalla M., Ibrahim I.M., Elfiky A.A. (2021). Caffeic acid derivatives (CAFDs) as inhibitors of SARS-CoV-2: CAFDs-based functional foods as a potential alternative approach to combat COVID-19. Phytomedicine.

[78] Guler H.I., Ay Sal F., Can Z., Kara Y., Yildiz O., Belduz A.O., Canakci S., Kolayli S. (2021). Targeting CoV-2 spike RBD and ACE-2 interaction with flavonoids of Anatolian propolis by in silico and in vitro studies in terms of possible COVID-19 therapeutics. Turk. J. Biol..

[79] Elfiky A.A. (2021). Natural products may interfere with SARS-CoV-2 attachment to the host cell. J. Biomol. Struct. Dyn..

[80] Pathak T., Pal S., Banerjee I. (2025). Cathepsins in cellular entry of human pathogenic viruses. J. Virol..

[81] Ulcakar L., Novinec M. (2020). Inhibition of Human Cathepsins B and L by Caffeic Acid and Its Derivatives. Biomolecules.

